# Thrombotic Microangiopathy in the Setting of Colorectal Cancer: A Therapeutic Challenge with a Bad Prognosis

**DOI:** 10.3390/hematolrep15010002

**Published:** 2023-01-04

**Authors:** Youssef Bouferraa, Yolla Haibe, Hanan Hamdan, Rami Mahfouz, Zaher Chakhachiro, Ali Shamseddine

**Affiliations:** 1 Department of Internal Medicine, Division of Hematology-Oncology, American University of Beirut Medical Center, Beirut 1107 2020, Lebanon; 2 Department of Pathology and Laboratory Medicine, American University of Beirut Medical Center, Beirut 1107 2020, Lebanon

**Keywords:** thrombotic thrombocytopenic purpura, plasmapheresis, colorectal cancer, anemia, thrombocytopenia

## Abstract

While most cases of thrombotic microangiopathic hemolytic anemias are idiopathic, some can occur in the setting of a malignancy. Differentiating both conditions is crucial to initiate the appropriate treatment. In this case report and literature review, we discuss the occurrence of a thrombotic microangiopathy in a 61-year-old male patient with a treatment-refractory metastatic colorectal cancer invading his bone marrow. Plasmapheresis does not constitute the mainstay of treatment in this setting, as targeting the primary disease is the ultimate management. Treating the condition of our patient has been challenging as multiple lines of treatments of his primary disease had been exhausted. The discrepancy in KRAs status obtained between PCR and later NGS offered a new treatment line with Cetuximab. In this article, we will discuss the different factors that differentiate between idiopathic and cancer-induced microangiopathy. We will emphasize on the fact that the treatment of the primary disease constitutes the most important step in the treatment of cancer-induced thrombotic microangiopathy. We will also raise several explanations to target the disagreement in KRAS status obtained by the different technical modalities.

## 1. Introduction

Thrombotic Thrombocytopenic Purpura (TTP) and Hemolytic Uremic Syndrome (HUS) are multisystem hematologic disorders characterized by the formation of platelet-rich thrombi in the small vessels, leading to microangiopathic hemolytic anemia, thrombocytopenia, and a wide range of possible organ damage. TTP and HUS are both “thrombotic microangiopathies”, which is the general class of disease manifestations they both belong to. Although low levels of ADAMTS13 are usually seen in TTP and not HUS, the two syndromes and their symptoms usually overlap, and the differentiation is not always clear-cut [1]. The occurrence of TTP/HUS can be life-threatening, especially in the setting of malignancies. As a result, identifying the causative agent and rapidly starting the appropriate treatment is of utmost importance. In this case report, we discuss a rare case of TTP/HUS in the setting of treatment-refractory colon adenocarcinoma with bone marrow metastasis.

## 2. Case

A 61-year-old male patient presented to our hospital complaining of epistaxis of 1-week duration, associated with easy bruising, occasional gingival bleeding, fatigue and decreased oral intake. The patient has a history of Stage IV KRAS mutated microsatellite stable (MSS) signet ring colon adenocarcinoma with metastasis to the bone, peritoneum, and retroperitoneum that was diagnosed 1 year prior to his presentation. He has received multiple lines of treatment in the past year. He first received 12 cycles of FOLFOX (5-Fluorouracil, oxaliplatin and leucovorin) and Bevacizumab during 5 months with CT scan showing a decrease in the size of the mass and metastatic deposits. Maintenance therapy was then established, and the patient received 6 cycles of 5-Fluorouracil and Bevacizumab with a further decrease in the size of the mass and deposits but an increase in the CEA levels from 6.6 to 12.9 ng/mL in 1 month. This same treatment was maintained for 2 more cycles. At this time, CEA levels continued to increase reaching 60 ng/mL in 5 weeks. As imaging also showed oligometastatic progression with new bone lesions, the patient was switched to FOLFIRI (Leucovorin, 5-Fluorouracil and Irinotecan) and Ramucirumab. He received 4 cycles of this treatment for 6 weeks. At this point, 3 weeks prior to his presentation, PET scan showed disease progression with increase in number and size of multiple liver, bone, and peritoneal metastasis. As a result, Next Generation Sequencing (NGS) was ordered on the initial biopsy and patient was started on FOLFOX and Aflibercept pending NGS results. He received 2 cycles, the last being 1 week prior to his presentation.

The patient was hemodynamically stable on presentation to the emergency department. Physical exam was unremarkable except for continuous epistaxis from his right nostril, associated with dry clots and crusts on the anterior septum. Blood workup was significant for a normocytic anemia [Hemoglobin 10.9 g/dL (Reference (Ref) 12–18 g/dL), Hematocrit 31% (Ref 37–54%), MCV 90.3 fl (Ref 80–100 fl)] with elevated reticulocyte count (8%, ref 0.2–2%), thrombocytopenia (35,600/cu.mm, Ref 150,000–400,000/cu.mm), elevated Lactate Dehydrogenase LDH (808 IU/L, Ref 110–265 IU/L) and low Haptoglobin levels (<0.1 g/L, Ref 0.3–2 g/). Bilirubin levels (T/D) were 1.6/0.5 mg/dL (Ref 0–1.2/0–0.3 mg/dL). Blood smear showed few acanthocytes, schistocytes, target cells and left-shifted granulocytes. Elevated creatinine (1.6 mg/dL, Ref 0.6–1.2 mg/dL) and SGOT (95 IU/L, Ref 0–50 IU/L) with normal SGPT (40 IU/L, Ref 0–50 IU/L) were also noted. Coagulation studies including PT-INR, PTT and fibrinogen were within normal limits. Direct and Indirect Coombs were negative.

**Figure 1:**
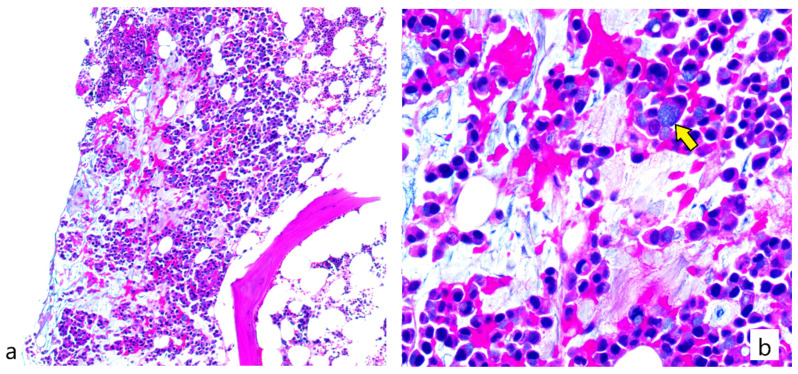
Bone marrow biopsy section showing (**a**) metastatic adenocarcinoma in the form of signet ring cells admixed within a background of bone marrow hemopoietic elements (**b**) Higher magnification showing signet ring carcinoma cells (pointed by yellow arrow) (H&E stain; (**a**): 100× magnification; (**b**): 400× magnification).

The patient was suspected to have thrombotic thrombocytopenic purpura/Hemolytic uremic syndrome (TTP/HUS) and was admitted for treatment of his disease. Unfortunately, ADAMTS-13 levels were not available at the time of presentation at our institution. He received 1 donor platelet transfusion given his low platelet count and epistaxis. Our differential included medication induced, idiopathic and cancer induced TTP/HUS. CEA levels were taken and showed a 2.5-fold increase from 489 ng/mL 2 weeks ago to 1239 ng/mL upon admission. The initial plan was to proceed with plasmapheresis which is known to be first line treatment for TTP/HUS. However, given the increase in CEA together with the leucoerythroblastic picture on peripheral blood smear, a bone marrow biopsy was performed and showed a focus of metastatic signet ring adenocarcinoma consistent with his primary disease (Figure 1). Consequently, plasmapheresis was deferred. However, given that primary TTP has not been completely ruled out, and given that steroids can transiently help with primary and cancer-induced TTP, he was started on Solumedrol 1 mg/kg IV daily with an increase of the dose up to 2 mg/kg IV daily in the next days. In parallel, labs showed an improvement in his platelet counts to 129,900/cu.mm and a gradual decrease in his reticulocyte count reaching 4.6% 9 days after his hospitalization. This improvement was only transient as platelet levels started dropping again, a finding that was associated with a parallel decrease in hemoglobin levels and increase in the reticulocyte count and LDH levels. At this time, NGS results showed wild type KRAS status contradictory to the results initially provided by Polymerase Chain Reaction (PCR).

As a result, the decision was made at the multidisciplinary tumor board to start the patient on single agent Cetuximab. Unfortunately, during his hospital stay, the patient developed dyspnea, oliguria, and worsening of his kidney injury. CT scan of the chest rules out a pulmonary embolism as a cause of acute dyspnea and was only significant bilateral small pleural effusion with sub segmental atelectasis. INR started to gradually increase. Patient also had an eight-fold elevation of his SGOT levels reaching 397 IU/L associated with an increase in bilirubin levels up to 15.5 mg/dL. Abdominal Ultrasound showed acute portal vein thrombosis. His kidney, liver and coagulation function further deteriorated until the patient suddenly developed dyspnea and bradycardia, went into cardiogenic shock, developed asystole, and passed away on day 13 of his admission.

## 3. Discussion

Our patient developed TTP/HUS late in the course of his refractory disease after failure of multiple lines of treatment. When he presented to the emergency department, the diagnosis of cancer-induced TTP was not clear yet, and idiopathic and drug-induced TTP were still on our differential list. As such, initial plans were to proceed with plasmapheresis. However, the increase in CEA levels and the leucoerythroblastic picture on the blood smear directed the medical team towards a bone marrow biopsy that in turn showed metastatic disease in the bone marrow. At this point, the diagnosis of cancer-induced microangiopathic hemolytic anemia was more supported and plasmapheresis was deferred. The aim of this case reports is to shed the light on the importance of early recognition of cancer-induced TTP/HUS in order to rapidly initiate anti-neoplastic therapy, which constitutes the first line therapy of this condition.

In our case, the patient’s situation was critical given the exhaustion of multiple lines of treatment of his disease. While a new treatment line was established as mentioned earlier, the initiation of the antineoplastic therapy was delayed given the above. Unfortunately, the patient developed systemic thrombosis and possibly disseminated intravascular coagulopathy. To note that the patient had a transient improvement during his hospital stay, likely secondary to the steroid effects. This is possible through the steroid-induced immunosuppression and decreased cytokine production [2]. However, this effect is only transient in the case of cancer-induced TTP as the inciting factor which is the primary malignancy is not eliminated unless anti-neoplastic therapy is initiated.

The occurrence of TTP in the setting of malignancy may be alarming, unexpected or can even go undiagnosed in the absence of high index of suspicion. Morton and George classify thrombotic microangiopathies in malignancies between treatment-induced and cancer-induced microangiopathic hemolytic anemias [3,4]. Several drugs including targeted therapy and chemotherapy can induce thrombotic microangiopathy through an immune-mediated or dose-dependent mechanism [3,4]. While the former is of a sudden onset and relies on antibody formation against the therapeutic agent, the latter is of a gradual onset and depends on the cumulative dose of the drug [3]. VEGF inhibition was associated with the onset of renal thrombotic microangiopathy. In a study performed by Eremina et al., selective VEGF gene deletion in the renal podocytes of mice models induced a thrombotic glomerular injury, attributing thus the occurrence of thrombotic microangiopathy to the local reduction in VEGF within the kidney environment [4]. While several reports of Bevacizumab-induced thrombotic microangiopathies are noted in the literature, we found only one published case report of a thrombotic microangiopathy in Aflibercpet-treated patients [4,5]. TTP in our patient may be in part attributed to VEGF inhibition.

On the other hand, Cancer-induced microangiopathic hemolytic anemia with bone marrow involvement is a stronger explanation of this patient’s presentation. In a retrospective review by Lechner and Obermeier, most cases of cancer-induced thrombotic microangiopathies between 1979 and 2012 occurred in gastric cancer patients, followed by breast, prostate and lung cancer respectively [6]. Few cases of TTP in colorectal cancer patients are reported (Table 1). The presentation of TTP/HUS is variable in colorectal cancer patients and can occur at different times during the course of the disease. While TTP was the presenting condition that led to the diagnosis in some cases, other cases presented with TTP along the course of their disease, at recurrence or even after surgery (Table 1).

Although the exact mechanism of cancer-induced thrombotic microangiopathies is not well understood, several possible explanations have been raised. Brain et al. attribute the red blood cell shearing to their direct contact with fibrin clots and tumor emboli within the blood vessels [15]. In addition, mucin produced by the tumor may directly contribute to endothelial cell dysfunction inducing formation of thrombi [16]. Moreover, it has been hypothesized that aggressive tumor growth within the bone marrow, along with secondary fibrosis and abnormal angiogenesis, may damage its vasculature leading to the release of Ultra Large Von Willebrand Factors (ULVWF) and thrombotic microangiopathy [6,17]. This might also be associated with the formation of autoantibodies against ADAMTS13 that usually cleaves VWF [6,17]. As a result, metastasis to the bone marrow is one of the main mechanisms involved in the development of thrombotic thrombocytopenic purpura in cancer patients. In an extensive review by Lechner and Obermeier, bone marrow metastasis was found in 81% of patients with cancer-induced thrombotic microangiopathies [6]. While this observation supports the important role of bone marrow metastasis in the pathophysiology of the disease, it also indicates that invasion of the bone marrow is not the only mechanism through which TTP and other thrombotic microangiopathies develop in those patients. The bone marrow biopsy in our patient showed signet-ring metastatic adenocarcinoma, favoring the fact that bone marrow invasion is the main trigger for the development of TTP in his case. In a review performed by Assi et al., bone marrow metastasis appeared to occur early in colorectal cancer, even at the microscopic level. They attribute the rarity of bone marrow metastasis as a first presentation of the disease to the decreased clinical need of BM studies in most cases, as well as the presence of other metastatic locations at the time of diagnosis that shift the attention from the bone marrow [18].

Differentiating between idiopathic and cancer-induced microangiopathic hemolytic anemia may not be straight forward. Table 2 summarizes some of the features that favor one over the other. This differentiation is an important step required to direct the appropriate treatment of the patient. The first line treatment for idiopathic TTP includes plasma exchange therapy and corticosteroids [19]. However, unlike idiopathic TTP, plasma exchange therapy does not constitute the mainstay of treatment of cancer-induced microangiopathic hemolytic anemia [3,6,20]. Treating the underlying malignancy with antineoplastic therapy has been shown to be the most beneficial intervention in the treatment of the disease [6,20]. However, and despite early recognition and initiation of anti-neoplastic therapy, these patients usually have widely disseminated disease that does not always respond appropriately to treatment. In a study by Francis et al., 9 out of 19 cancer-induced TTP patients received chemotherapy, and 5 achieved complete remission [21]. Other studies even reported lower success rates, and this was attributed to the poor prognosis of the disseminated malignancy, even in the absence of TTP [9]. This being said, when cancer-induced microangiopathic hemolytic anemia is suspected, plasma exchange therapy should be carefully considered not only to avoid unnecessary treatment but also to prevent delays in diagnosis and treatment initiation as well as complications of this therapy [22]. In fact, plasma exchange therapy can be associated with multiple fatal and non-fatal complications including sepsis secondary to bacteremia, central venous line thrombosis, multi-structure perforations secondary to catheter insertion and anaphylaxis, which can be prevented in the setting of unnecessary catheter use [3]. While advances in the medical technology have decreased the risks of those complications, such side effects still exist and avoiding unnecessary treatments and complications should always be considered.

Our patient presented with metastatic disease progression after failure of multiple treatment lines. However, the contradictory results by NGS showing wild type KRAS status paved the path towards a new treatment line with Cetuximab. In fact, KRAS status is important in assessing the response of colorectal cancer to EGFR-targeting agents. PCR and NGS provided discordant results in our case. NGS, as in any other sequencing, may fall into an allelic dropout during PCR amplification or, more commonly, sample variation in tumor load in cases of exhausted material referred out for analysis. Moreover, we cannot preclude the possibility of having a change in KRAS Status from mutant to wildtype after 1 year of treatment [25]. This change of status could be in part explained by the loss of RAS mutation previously reported in heavily pretreated patients, allowing a higher response rate to Cetuximab [25].

Even with treatment initiation, the prognosis of cancer-induced microangiopathic hemolytic anemia is very poor and most patients die shortly after diagnosis [21]. As a result, once a diagnosis of cancer-induced TMA has been established, an emergent decision about the initiation of aggressive anti-neoplastic treatment or plan for palliative care should be made. Palliative supportive measures are usually established to target the symptomatic complications of TTP. Palliative radiation therapy can be considered on a case-by-case basis in an attempt to relieve symptoms and improve comfort [26]. In addition, for patient with active bleeding, we do not withhold platelet transfusions for fear of increasing the risk of thrombosis [27]. Individuals with neurologic findings or cardiac dysfunction should be evaluated by neurology and cardiac consultants respectively, especially if the symptoms are interfering with their quality of life. The use of analgesic therapy should be individualized to the needs of every patient [28]. Moreover, dyspnea, whether attributed to physical or psychological origin should be adequately managed in patients with cancer induced TTP. Finally, alleviations of the psychological, sociological and spiritual problems constitute essential components of the palliative treatments of those patients [28].

Our approach would have been different if palliative treatment as detailed above has been discussed with the patient. This patient has already a stage IV metastatic disease with a poor prognosis even without the development of thrombotic microangiopathy. As such, it would have been appropriate to symptomatically treat the patient to ensure appropriate pain control, restful breathing, and psychologic support. Discussion of goals of care and possibly transition to comfort care could have made some of the unnecessary suffering avoidable.

## 4. Conclusions

In conclusion, physicians should have a low threshold in diagnosing cancer-induced thrombotic microangiopathies. Although not routinely performed, a bone marrow biopsy should be considered, especially in the presence of thrombocytopenia and nucleated RBCs on blood smears. The presence of bone marrow metastasis usually supports the diagnosis. Plasmapheresis, if considered, should not delay the primary treatment with antineoplastic therapy. In this case report, the patient presented with TTP very late in the course of his refractory disease raising the question about the possible treatments of cancer-induced microangiopathic hemolytic anemias presenting at disease progression after multiple lines of treatment were already administered.

## Figures and Tables

**Table 1:** Case reports of thrombotic microangiopathies (TTP/HUS) in the setting of colorectal cancer.

Identification	Age, Gender	Histology	Bone Marrow	TTP Onset Relative to Cancer Diagnosis	Treatment	Outcome of TTP/HUS	Survival since TTP (Months)
Lee et al., 2004 [7]	67, M	Poorly differentiated Adenocarcinoma	Fibrosis	At diagnosis	Plasmapheresis and plasma exchange	No response	
					FOLFOX-4	Complete remission	4.5 (Until date reported)
Majhail et al., 2002 [8]	66, F	Moderately differentiated adenocarcinoma	N/A	At diagnosis	Prednisone and plasma exchange (for a total of 11 weeks) with left hemicolectomy at week 5)	Complete remission at week 5	9 (until date reported)
Oberic et al., 2009 [9]	54, F	Adenocarcinoma	Fibrosis and metastatic infiltration	At diagnosis	High dose plasma infusion + steroids + FOLFOX-5	Death due to coma	2 days
Park et al., 2018 [10]	76, F	Poorly differentiated adenocarcinoma	Metastatic infiltration	At recurrence	Plasma exchange	Death	10 days
Usami et al., 2008 [11]	60, F	Moderately differentiated adenocarcinoma	N/A	At diagnosis	Distal colectomy	Complete remission	14 days (Until date reported)
Ducos et al., 2014 [12]	43, M	N/A	N/A	30 days after diagnosis	N/A	Death	N/A
Robson et al., 1997 [13]	64, F	Moderately differentiated adenocarcinoma	N/A	After Right hemicolectomy	Plasma exchange with FFP	Complete remission	11 days (until date reported)
Lohrmann et al., 1973 [14]	63, M	Adenocarcinoma	Increased erythropoiesis and megakaryopoeisis	2 months after diagnosis	N/A	N/A	N/A

**Table 2:** Differences between cancer-induced and idiopathic TTP.

	Cancer-Induced TTP	Idiopathic TTP
Age [23]	Usually older	Usually younger
Previously diagnosed cancer [3]	Usually present	Usually absent
Onset [3]	Gradual	Sudden
Kidney injury [3]	Common	Uncommon
LDH levels [3]	Very High	High
Nucleated RBCs on smear [19]	More common	Less common
Back pain [3,20,24]	More common	Less common
Dyspnea and pulmonary infiltrates [3,20,24]	More common	Less common
Risk of late-onset coagulation abnormalities and DIC [3]	High	Low
ADAMTS13 levels [3]	Normal or slightly decreased	Decreased

## Data Availability

No new data were created or analyzed in this study. Data sharing is not applicable to this article.
